# Negative Effekte der COVID-19-Maßnahmen auf die Versorgung depressiv Erkrankter: Ergebnisse einer repräsentativen Bevölkerungsbefragung

**DOI:** 10.1007/s00115-021-01148-3

**Published:** 2021-06-17

**Authors:** Hanna Reich, Andreas Czaplicki, Christian Gravert, Ulrich Hegerl

**Affiliations:** 1 Forschungszentrum Depression der Stiftung Deutsche Depressionshilfe, Klinik für Psychiatrie, Psychosomatik und Psychotherapie, Johann-Wolfgang-Goethe-Universität, Frankfurt am Main, Deutschland; 2 Stiftung Deutsche Depressionshilfe, Leipzig, Deutschland; 3 Deutsche Bahn AG, Berlin, Deutschland; 4 Johann Christian Senckenberg Distinguished Professorship, Klinik für Psychiatrie, Psychosomatik und Psychotherapie, Johann-Wolfgang-Goethe-Universität, Frankfurt am Main, Deutschland; 5 Universitätsklinikum Frankfurt, Klinik für Psychiatrie, Psychosomatik und Psychotherapie, Stiftung Deutsche Depressionshilfe, Heinrich-Hoffmann-Str. 10, 60528 Frankfurt am Main, Deutschland

Akteure in der Politik, im Medizinbetrieb und in vielen anderen Bereichen haben eingreifend und teils rasch und zielgerichtet auf die Bedrohungen durch die COVID-19-Pandemie reagiert. Mit Blick auf die Situation in anderen Ländern wurden die getroffenen Maßnahmen meist als erfolgreich, wenn nicht gar vorbildlich dargestellt und dabei auf die Infektionszahlen und COVID-19-assoziierten Todesfälle verwiesen. Diese Zahlen allein sind jedoch für eine Erfolgsbeurteilung nicht ausreichend, da dadurch die „Nebenwirkungen“ der getroffenen Maßnahmen vernachlässigt werden. Entscheidend ist die Frage nach dem Verhältnis zwischen dem Ausmaß an Leid und Tod, das durch die getroffenen Maßnahmen einerseits verhindert und andererseits verursacht wird. Berichte über gravierende negative gesundheitliche Folgen dieser Maßnahmen häufen sich. In Deutschland wie auch in anderen Ländern wurde nach Ausbruch der Corona-Pandemie über eine verzögerte medizinische Kontaktaufnahme von Patient:innen nach Herzinfarkt oder Schlaganfall verbunden mit deutlich erhöhtem intrahospitalem Sterberisiko berichtet [2, 8, 9]. Auch die Versorgung von Patient:innen mit Suchterkrankungen war bedingt durch monatelange Schließungen ambulanter Drogenhilfeeinrichtungen erschwert [4].

Mit dieser Arbeit soll ein Beitrag zu der dringend benötigten Diskussion der Kosten-Nutzen-Abschätzung bzgl. der Maßnahmen zur Eindämmung des Infektionsgeschehens geleistet werden, indem aus Betroffenensicht deren Einfluss auf die medizinische und psychotherapeutische Versorgung depressiv Erkrankter dargestellt wird.

In einer bevölkerungsrepräsentativen Umfrage der deutschen Wohnbevölkerung im Alter von 18 bis 69 Jahren wurden 5178 Personen (*n* = 2660 weiblich) im Zeitraum vom 26.06.2020 bis 08.07.2020 durch das zertifizierte Befragungsunternehmen (ISO 26362) Respondi AG online befragt (Teilnahmequote: 31,3 %). Die Stichprobe stellt eine mehrfach geschichtete Quotenauswahl anhand der verschränkten Merkmale Geschlecht, Alter, Bundesland/Bundesländergruppe entsprechend der aktuellen Bevölkerungsfortschreibungen des Statistischen Bundesamts dar. Alle Teilnehmenden waren im Access Panel registriert und haben ihre schriftliche Einwilligungserklärung zur Teilnahme gegeben. Eine Aufwandsentschädigung erfolgte in Form von Punkten (Gegenwert: 1 €), die für Internetkäufe eingesetzt werden können. Einschränkungen in der allgemeinen medizinischen Versorgung wurden für den Befragungszeitraum („aktuell“) und retrospektiv für die erste Lockdownphase („4 Wochen des Lockdowns“) folgendermaßen erfragt: „Durch die Maßnahmen gegen Corona sind bei mir oder einem nahen Angehörigen wichtige Arzttermine ausgefallen/wichtige medizinische Behandlungen nicht durchgeführt worden/die Behandlungsmöglichkeiten beeinträchtigt gewesen“ (Likert-Skala 1–4). Teilnehmende mit diagnostizierter Depression (*n* = 1,094 „Ja, bei mir ist bereits einmal die Diagnose Depression gestellt worden“; davon *n* = 197 „Ich befinde mich aktuell in einer depressiven Phase“) wurden zu spezifischen Auswirkungen auf die Versorgung ihrer Depressionserkrankung befragt (ja/nein), die Datenauswertung erfolgte mit Stata SE15.1 (α = 0,05).

Mehr als ein Drittel der befragten Allgemeinbevölkerung gab an, während des ersten Lockdowns Einschränkungen der medizinischen Versorgung bei sich selbst oder einem nahen Angehörigen erlebt zu haben; auch zum Befragungszeitpunkt im Sommer berichtete dies immer noch fast ein Viertel aller Befragten (Tab. 1). Unter den Teilnehmenden mit einer aktuellen depressiven Krankheitsphase lagen die berichteten Einschränkungen mit 56 % (Lockdown) bzw. 42 % (Juni/Juli) nochmals höher. Hierbei gaben außerdem 13 % der Betroffenen an, selbst ambulante Termine abgesagt zu haben, weil ihnen die Situation zu unsicher gewesen sei. Jeder fünfte männliche Teilnehmer mit diagnostizierter Depression berichtete von Terminausfällen beim Facharzt, das Risiko für Ausfälle war damit fast doppelt so hoch wie bei Frauen (Tab. 2). In Bezug auf psychotherapeutische Behandlungen war das Risiko von Terminausfällen für die Geschlechter vergleichbar, jedoch berichteten jüngere Erwachsene mit einer 1,6-fach erhöhten Wahrscheinlichkeit von Ausfällen psychotherapeutischer Termine im Vergleich zu Erwachsenen mittleren Alters. Dass über 60-Jährigen seltener über Ausfälle von Psychotherapiesitzungen oder Selbsthilfegruppen berichten, mag Ausdruck dessen sein, dass derartige Versorgungsangebote älteren Menschen nur selten gemacht werden.

**Table 1:** 

		Während des 1. Lockdowns				Aktuell (Juni/Juli 2020)				Signifikanz Zeiteffekt
		*n*	%w	[95 %-CI]		*n*	%w	[95 %-CI]		
Lebenszeitdiagnose Depression	Aktuelle depressive Episode (*n* = 197)	110	**56,3**	49,2	63,0	83	**42,4**	35,7	49,5	*p* < 0,001
	In Remission (*n* = 897)	352	39,4	36,2	42,6	221	24,7	22,0	27,7	*p* < 0,001
Allgemeinbevölkerung (*n* = 4084)		1413	34,8	33,3	36,3	889	22,0	20,7	23,3	*p* < 0,001
Signifikanz Gruppeneffekt		–	*p* < 0,001	–	–	–	*p* = 0,004	–	–	–

Nach Alter, Geschlecht und Bundesland gewichtete Prozentangaben (%w [95%iges Konfidenzintervall])

Die Antwort „trifft zu“ oder „trifft sehr zu“ auf mindestens eins der drei Items („Durch die Maßnahmen gegen Corona sind bei mir oder einem nahen Angehörigen wichtige Arzttermine ausgefallen/wichtige medizinische Behandlungen nicht durchgeführt worden/die Behandlungsmöglichkeiten beeinträchtigt gewesen“) wurde als Einschränkung gewertet

Test der Gruppeneffekte mit univariaten ANOVAs, Test der Zeiteffekte mittels gepaarter t‑Tests

Signifikante Unterschiede sind **fett** hervorgehoben

**Table 2:** 

Ausfall von Terminen			*n/Gesamt*	%w	[95 %-CI]		OR	[95 %-CI]		*p*
Hausarzt	–	*–*	112/1094	10,3	8,6	12,2	*–*	–	–	–
	Geschlecht	W (= Ref.)	59/639	9,2	7,2	11,7	*1,00*	–	–	–
		M	53/455	11,7	9,0	15,0	1,30	0,88	1,93	0,193
	Alter	18–29 J	10/135	7,7	4,1	13,8	0,72	0,36	1,43	0,344
		30–59 J. (= Ref.)	76/723	10,5	8,5	13,0	*1,00*	–	–	–
		60–69 J	26/236	11,0	7,6	15,7	1,07	0,66	1,71	0,790
Facharzt	–	–	171/1094	15,8	13,8	18,2	*–*	–	–	–
	Geschlecht	W (= Ref.)	78/639	**12,2**	**9,9**	**15,0**	*1,00*	–	–	–
		M	93/455	**20,7**	**17,2**	**24,7**	**1,89**	**1,36**	**2,64**	**<** **0,001**
	Alter	18–29 J	24/135	18,6	12,7	26,3	1,28	0,78	2,08	0,324
		30–59 J. (= Ref.)	113/723	15,7	13,2	18,6	*1,00*	–	–	–
		60–69 J	34/236	14,4	10,5	19,6	0,94	0,62	1,43	0,764
Psychotherapeut	–	–	134/1094	12,4	10,5	14,5	*–*	–	–	–
	Geschlecht	W (= Ref.)	74/639	11,6	9,3	14,3	*1,00*	–	–	–
		M	60/455	13,3	10,5	16,8	1,17	0,81	1,70	0,396
	Alter	18–29 J	26/135	**19,4**	**13,5**	**27,1**	**1,65**	**1,02**	**2,68**	**0,042**
		30–59 J. (= Ref.)	93/723	**12,8**	**10,6**	**15,5**	*1,00*	–	–	–
		60–69 J	15/236	**6,4**	**3,8**	**10,3**	**0,46**	**0,26**	**0,82**	**0,008**
Geplanter stationärer Aufenthalt	–	–	35/1094	3,2	2,3	4,5	*–*	–	–	–
	Geschlecht	W (= Ref.)	19/639	3,0	1,9	4,7	*1,00*	–	–	–
		M	16/455	3,5	2,2	5,7	1,15	0,59	2,27	0,680
	Alter	18–29 J	5/135	**3,7**	**1,5**	**8,8**	0,97	0,37	2,55	0,947
		30–59 J. (= Ref.)	28/723	**3,9**	**2,7**	**5,6**	*1,00*	–	–	–
		60–69 J	2/236	**0,9**	**0,2**	**3,4**	**0,21**	**0,05**	**0,91**	**0,037**
Selbsthilfegruppe	–	–	67/1094	6,2	4,9	7,8	*–*	–	–	–
	Geschlecht	W (= Ref.)	41/639	6,4	4,7	8,6	*1,00*	–	–	–
		M	26/455	5,9	4,0	8,5	0,88	0,53	1,47	0,634
	Alter	18–29 J	6/135	**4,4**	**2,0**	**9,6**	0,58	0,24	1,38	0,214
		30–59 J. (= Ref.)	53/723	**7,4**	**5,7**	**9,5**	*1,00*	–	–	–
		60–69 J	8/236	**3,4**	**1,7**	**6,6**	**0,43**	**0,20**	**0,92**	**0,030**

Nach Alter, Geschlecht und Bundesland gewichtete Prozentangaben (%w [95%iges Konfidenzintervall]) und Odds Ratios (*OR*) aus multiplen logistischen Regressionen (für Geschlecht und Alter adjustiert)

Von *n* = 1094 Personen mit diagnostizierten depressiven Erkrankungen waren *n* = 197 aktuell depressiv, *n* *=* *897* remittiert depressiv

Signifikante Unterschiede sind **fett** hervorgehoben

Die Referenzkategorien sind mit (= Ref.) gekennzeichnet

Hochgerechnet betrafen die hier erfragten Einschränkungen in der medizinischen Versorgung im ersten Jahr der Pandemie ca. 2 bis 3 Mio. Menschen mit affektiven Erkrankungen [3]. Diesen unter einer schweren, oft auch lebensbedrohlichen Erkrankung leidenden Menschen wurde eine schlechtere Versorgung ihrer Erkrankung zugemutet, um das Infektionsgeschehen zu verlangsamen oder Versorgungskapazitäten für COVID-19-Infizierte zu schaffen. Ähnliche Befunde zu einem Rückgang der Versorgung psychischer Erkrankungen liegen auch aus Großbritannien vor [5]. Männliche Befragungsteilnehmer und jüngere Erwachsene berichteten in der vorliegenden Befragung von stärkeren Einschränkungen als die jeweiligen Referenzgruppen (Tab. 2). Gleichzeitig zeigte eine longitudinale Studie, dass das Stresserleben bei jungen Erwachsenen sowie depressive und Angstsymptome bei jungen Frauen im Besonderen im Zuge der Pandemie zunahmen [7]. Junge Erwachsene waren damit sowohl mit erhöhten Belastungen ihrer psychischen Gesundheit als auch mit stärkeren Einschränkungen in der psychotherapeutischen Versorgung während der COVID-19-Pandemie konfrontiert. Eine mangelhafte Versorgung depressiver Erkrankungen junger Erwachsener kann mit hohen, langfristigen Kosten für Individuum und Gesellschaft einhergehen [1]. Die Sicherstellung zeitnaher, leitliniengerechter Behandlungen depressiver Erkrankungen sollte daher auch in Anbetracht ihres gesamtgesellschaftlichen Nutzens in den aktuellen politischen Entscheidungen stärker Eingang finden.

Bei der vorliegenden Arbeit handelt es sich um Analysen von Daten einer sozialwissenschaftlichen Befragung. Eine methodische Einschränkung betrifft Fragen, die sich auf den ersten Lockdown beziehen, da diese retrospektiv im Juni/Juli gestellt wurden. Zudem erfolgte bei der Erfragung allgemeiner Einschränkungen (Tab. 1) keine Differenzierung zwischen Erkrankten selbst und Angehörigen; eine getrennte Auswertung ist an dieser Stelle somit nicht möglich.

Eine nicht nur auf das Infektionsgeschehen verengte Sicht macht deutlich, dass die Implementierung besonders strenger Maßnahmen in der COVID-19-Pandemie nicht „Auf Nummer sicher gehen“ bedeutet und dass es auch nicht um Gesundheit vs. Ökonomie geht, sondern um die richtige Balance zwischen Nutzen und Risiken der Maßnahmen. Eine Gesamtbilanzierung sollte weitere Aspekte beinhalten, die hier nicht betrachtet wurden. Diese umfassen neben ökonomischen und freiheitsrechtlichen Aspekten auch die Frage, ob sich die in einigen Bevölkerungsgruppen beobachteten Zunahmen von depressiven und Angstsymptomen [7] sowie des Alkoholkonsums [6] langfristig in einer Veränderung von Prävalenzraten psychischer Erkrankungen abbilden. Telemedizinische und digitale Behandlungsangebote haben während der Pandemie noch einmal an Bedeutung gewonnen. Einstellungen zu und Nutzung von diesen Angeboten sollten in zukünftigen Studien untersucht werden.

## Fazit für die Praxis

- Die Maßnahmen zur Verlangsamung des COVID-19-Infektionsgeschehens gingen für depressiv erkrankte Menschen mit einem Verlust an Versorgungsqualität (ausgefallene ambulante Behandlungstermine, abgesagte stationäre Behandlungen) einher.
- Bei jungen und bei männlichen Erwachsenen war die Rate ausgefallener ambulanter Termine besonders hoch.
- Auch während der COVID-19-Pandemie bleibt das Recht von Patient:innen auf eine leitlinienkonforme Behandlung ihrer depressiven Erkrankung unverändert bestehen.
